# “A Doubt is at Best an Unsafe Standard”: Measuring Sugar in the Early Bureau of Standards

**DOI:** 10.6028/jres.112.004

**Published:** 2007-02-01

**Authors:** David Singerman

**Affiliations:** National Institute of Standards and Technology, Gaithersburg, MD 20899

**Keywords:** Bates, Frederick, Bureau of Standards, customs, instruments, measurement, metrology, optics, polarimetry, saccharimetry, standards, sugar

## Abstract

In 1900, measuring the purity of sugar was a problem with serious economic consequences, and Congress created the Bureau of Standards in part to create accurate standards for saccharimetry. To direct the Polarimetry Section, Director Stratton hired the young chemist Frederick Bates, who went on to make significant contributions to the discipline of sugar chemistry. This paper explores four of Bates’s greatest accomplishments: identifying the error caused by clarifying lead acetate, inventing the remarkable quartz-compensating saccharimeter with adjustable sensibility, discovering the significant error in the prevailing Ventzke saccharimetric scale, and reviving the International Commission for Uniform Methods of Sugar Analysis to unify the international community of chemists after the tensions of World War One. It also shows how accomplishments in saccharimetry reflected the growing importance and confidence of the Bureau of Standards, and how its scientific success smoothed the operation of American commerce.

## 1. What is Sugar?

Sugar is the most common sweetener in the world. It is a carbohydrate, sucrose, a disaccharide formed by joining carbon atom 1 of a glucose molecule with carbon atom 2 of a fructose molecule. Sucrose is a key product of photosynthesis and thus makes its way into almost every part of the human diet; it is also nearly unique in that humans consume it in unadulterated crystalline form.

Since about 8000 B.C.E., humans have cultivated sugar cane (Saccharum officinarum) to extract sucrose from its juice. In a warm, moist climate, sugar cane will grow an inch a day for weeks, and when ripe cane contains the most juice it is almost two inches in diameter. The cane must be chopped, ground, and immersed in water quickly, before the juice has a chance to rot. The water is then heated and evaporated, leaving the solution supersaturated with sucrose. As the water cools, the sugar begins to crystallize, leaving behind a thick, dark substance called molasses, which cannot be crystallized further but is a cheap way to sweeten foods (or to make rum).

The crystals left by evaporation are called “raw” sugar because they still contain little bits of cane and other impurities. They are now stable, however, and very economical to transport. The remaining steps, which repeatedly heat and cool the raw sugar as it progresses towards the “refined” state, can occur far from the cane fields. In the modern world, raw sugar has often crossed national borders in search of refineries [1].

## 2. A Brief History of Sugar

Cane sugar first made its way to Europe in the early years of the second millennium, but for centuries, only the very rich could afford to import it. Most Europeans sweetened their bland diets with honey, and it was this substance to which “sweetness” was attached; sugar was just another “spice” to be used in combination with others. One fourteenth-century recipe for “Oyster in gravy Bastard,” for example, instructed the cook to combine sugar with shellfish, ale, bread, ginger, and saffron [1].

Those who could afford sugar could afford a lot of it, and sugar had purposes other than flavoring. Kings and nobles often finished their feasts by presenting fantastically elaborate sugar sculptures. The size, scale, and complexity of each “subtlety” reflected the power and wealth of the noble who served it. Some depicted Biblical stories or historic battles, while others dazzled with the virtuosity of an artisan—a sailing ship with working cannons, a scale model of Oxford University, or a stag with an arrow sealing a “wound” that “bled” wine [1].

Just as they did with other spices, the mercantilist empire planners of Europe dreamed of possessing a limitless supply of sugar. The Spanish, Portuguese, French, and English all established empires in the New World in order to cultivate cane and thus corner the market for the commodity. As supply from the colonies affected the European market, the price drifted gradually downward, and the new merchant classes began to purchase and use sugar in small amounts. While they did create some smaller subtleties of their own, the middle class found more use for sugar in pastries and sweet desserts. Within the classes below the aristocracy, sugar became a demonstration of social status, but the price of sugar had not yet fallen far enough to let ordinary citizens enjoy it [1].

The European governments, following mercantilist theory, maintained high tariffs and restrictive trade policies on sugar from other empires. The resulting high prices gave colonial sugar planters wealth that rivaled that of royalty. Ironically, as historian Eric Williams explains in *Capitalism and Slavery*, the planters’ own riches ended their dominance of the economic system. The European banks in which the planters deposited their wealth invested in the beginnings of the Industrial Revolution [2].

That economic and social upheaval transformed the traditional workday, in which patterns of work had followed the sun, into one in which the needs of machines and the ticking of clocks regulated the rhythm of life. Factory employees no longer enjoyed meals at home but instead now ate at the site of their labor. Food was no longer hot or fresh, and many diets did not provide enough calories to power tough industrial labor. As the anthropologist Sidney Mintz writes in *Sweetness and Power*, the English working class turned to a new beverage for energy and heat.

The Royal East India Company began to import tea leaves from China in the late seventeenth century. The English, long fans of ale, quickly developed a taste for the new drink; the Company’s imports rose from twenty thousand pounds in 1700, to over two million pounds in 1800; and smuggling, the government unhappily estimated, brought in just as much [1]. Tea could be bitter, however, so the English became consumers not of tea “neat” but of tea with sugar. For the worker, sweetened tea proved a potent concoction; as sugar’s calories kept them fueled, while its caffeine kept them awake. As factory life altered the lives and eating habits of more and more workers, they began to demand sugar at a lower price and in higher quantities than the mercantilist system could supply [1].

In part because of pressure from sugar-hungry industrialists, tariff policy began to change during the late eighteenth century. Backed by philosophers and economists who were developing the new theory of free trade, factory owners argued that high tariffs prohibited the most efficient use of economic resources, including sugar. Colonial planters desperately tried to use their remaining political power to maintain the protection they enjoyed from foreign competition, and they complained that industrialists had betrayed fellow aristocrats to placate the working poor that toiled in their new factories [1].

They were right. Manufacturing interests had gained influence with economic power, and used it to convince Parliament to make sugar available at prices that working Britons could afford. At the same time, the British government decided that levying a low tariff on a large amount of sugar would generate more revenue than a high one, on a relatively small amount. The result was a low price of sugar that made its use possible, not only in tea, but also throughout the ordinary English diet. What had previously been an aristocratic delicacy had now become an irreplaceable source of calories and satisfaction for the middle and working classes.

As the United States began to industrialize, slowly at first, in the antebellum years, then much more rapidly after 1865, it also developed a noticeable appetite for sugar. In the last quarter of the nineteenth century, as industrialization transformed the society, America’s per-capita consumption of sugar skyrocketed. Just as importantly, the growing Federal government also came to rely on sugar as it took in enormous sums from customs duties on sucrose. Thus measuring sugar’s purity at the border became a problem of immense political and scientific importance.

## 3. The Sugar Tariff in America Before the Bureau of Standards^1^

Parliament made sure that the British sugar colonies enjoyed a protected market throughout the whole empire, so in 1733, London imposed a tax on every gallon of foreign molasses flowing to the North American colonies. In the decade before the Revolution, Parliament passed the Sugar Act, which brought in ninety-seven percent of the tax revenue collected in the American colonies, by 1774 [3]. By 1789, though, it had fallen to the recently independent American government to levy its own tariffs. The new government distinguished between raw and refined sugar and continued to do so for the next century and a half.

America had different economic interests at stake than Britain. Whereas Parliament wanted to shield its colonial planters from foreign competitors, Congress had few growers to protect. Instead, it was the industries that processed sugar—the refiners and rum distillers—that needed tariff protection from their rivals. Thus, refined sugar was taxed at a higher rate than raw. Thus, the tariff on raw sugar existed almost entirely to make money for the government, not to protect any domestic interest or constituency.

Inspectors generally weighed a sample to calculate its density, which was presumed to have a direct relationship to the proportion of sucrose. By the eighteen-thirties, however, importers began to increase the sucrose content of “raw” sugars and molasses, altering the already questionable connection between density and purity and forcing the Treasury Department, which oversaw the Customs Service, to find a new way of determining how much to tax a given shipment. The Secretary of the Treasury, John Spencer, eventually turned to the Office of Weights and Measures, who, in turn, appointed Richard McCulloh, professor of chemistry at Jefferson College in Philadelphia, to report on other methods of saccharimetry. The first formal relationship between the Customs Service and a standardization agency took place when Secretary Spencer ordered inspectors at major ports to submit samples to the college’s laboratory to guard against fraud.

McCulloh concluded that the specific gravity of a sample had no connection to its purity. He also, in a series of reports between 1845 and 1848, brought to the government’s attention, the “polarimeter,” an invention of the French physicist Jean-Baptiste Biot. The polarimeter could measure the concentration of a known optically active material by its rotation of the plane of polarization of light.^2^ However, as Deborah Warner of the Smithsonian Institution recently pointed out, the scientific and business communities split on whether a polarimeter would provide ample opportunity for fraud, how much scientific training was needed for its use, and how accurate it was in theory and in practice.

In 1861, therefore, Congress decided to adopt the Dutch method of measuring the purity of sugar by its color, according a scale standardized by Amsterdam merchants. But color, like density, proved unrelated to the sugar content. Soon, importers began to manipulate their shipments’ hue to take advantage of the Dutch scale. The Treasury was stuck. On one hand, if it declared that the “true” color of the sugar was that obtained, as one Secretary declared—only “by the ordinary processes of manufacture” and not by any later additives—then testers would have to use another method (such as a polarimeter) to determine how sugary a sample of syrup was. But when the Department relied on a method that was neither scientifically accepted nor expressly permitted by statute, importers had an easy case in court. On the other hand, if the Treasury decided to judge only by the sugar’s apparent color, importers would easily defraud the American public out of millions of dollars.

Despite numerous hearings that revealed the financial importance of the problem, Congress failed to produce any legislation that dictated the method to be used by the Customs Service. Frustrated, the Secretary tried to let each inspector do as he pleased. The Supreme Court, however, decided in 1882 that Congress held sole authority over the method of testing. It ordered the Treasury to refund millions in duties and return to the Dutch standard.

Immediately following that decision, however, the House Ways and Means Committee specifically appointed a commission to revise the tariff system. After hearing from both scientific and industrial witnesses, the members recommended that the polarimeter be adopted. Simultaneously, the National Academy of Sciences confirmed that using the device was as accurate as any other method, if not more so. Finally, in 1883, Congress acceded to the polarimeter’s employment by the Customs laboratories.

By this point, the growing American industrial working class demanded sugar as if it were a necessity of life, and the tariff had become an indispensable source of money for the Federal government. Though Congress had enacted a small income tax during the Civil War, the government paid for its expenses with excise taxes (mostly on vices like alcohol or tobacco) through the sale of land and through tariffs on imports. The sugar tariff, which alone accounted for between one-fifth and one-sixth of all revenue, was the largest single source of funds [3].

**Fig. 1:**
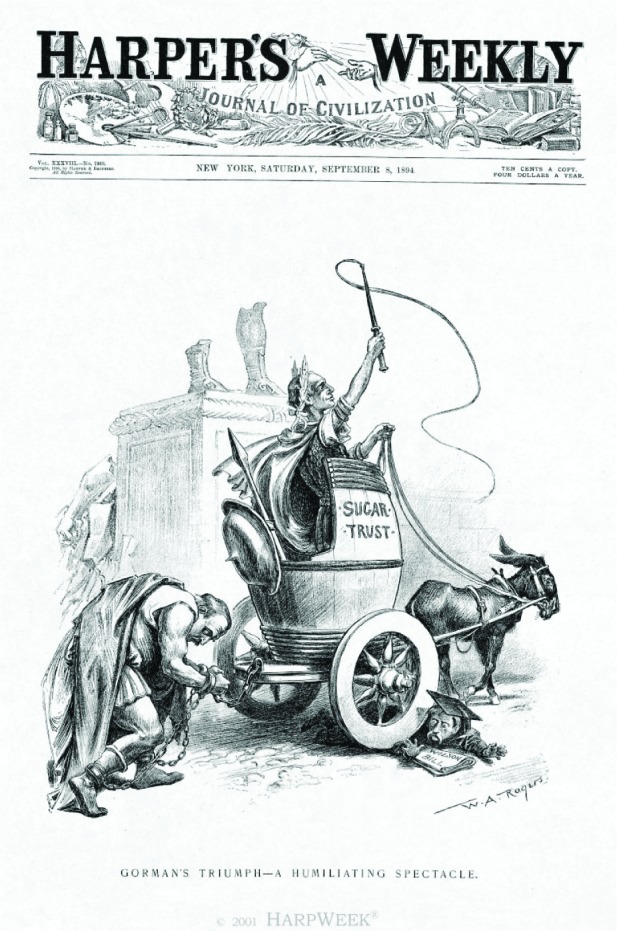
The cover of Harper’s Weekly from September 8, 1894, entitled “Gorman’s Triumph – A Humiliating Spectacle,” shows the victorious sugar trust pulling a defeated President Cleveland. Congressman Wilson and his low-tariff bill are crushed under the wheel of the chariot. (Harper’s Magazine, www.harpweek.com)

Between 1880 and 1890, annual consumption of sugar rose from forty-two to fifty-two pounds per American. The world price of sugar fell during roughly the same period, from 10 cents per pound in 1870, to 3.2 cents in 1884. But in 1890, Congress passed the McKinley Tariff, which placed both raw sugar and molasses on a list of duty-free products. To protect the domestic refining industry, refined sugar was still taxed at half a cent per pound. Lifting the tariff led to extraordinary growth in consumption—in 1891, each American used an astonishing 66.3 pounds of sugar [3]. However, the recession of 1893 took a heavy toll on other sources of income for the government, and in 1894, Congress passed the highly unpopular Wilson-Gorman Tariff (Fig. 1), which again imposed a small duty on raw sugar, and raised it, again, in 1897 [4].

For the Treasury and for the refining and importing industries, therefore, an enormous amount of money rested on the success or failure of the polarimetric method of testing sugar. It was troubling, however, that the adoption of the polarimeter did not end the flood of costly challenges to Customs decisions, nor did it impose uniformity on laboratories in different cities. By the late eighteen-eighties the appraisers in Boston and New York disagreed seriously enough that the Treasury hired investigators to look into the discrepancy.

In July of 1890 the Secretary of the Treasury, William Windom, sent Congress the final report on the matter. The original complaint had been filed by Massachusetts merchants who accused the examiner in New York, Dr. Edward Sherer, of making “improper and corrupt” measurements that, because they resulted in lower tariffs for importers, had stolen business away from Boston. Sherer had already been dismissed once before, though he was reinstated when evidence came to light that suggested his equipment had been tampered with. But when the agents tried to discover whether Dr. Sherer had intentionally reported lower values of sugars to aid his city’s importers, they were baffled.

The investigators first thought the problem might involve the quartz plates used to calibrate saccharimeters. Dr. Leary, the Boston examiner, had a certain plate marked “99 ½,”—that is, it would read 99 ½ degrees on a properly calibrated instrument—and the agents decided to have it tested for accuracy. But a test turned out to be more difficult than they imagined, because no one could agree on what “99 ½” meant. Dr. Sherer thought that the fraction actually was a decimal, so that “99 ½” actually lay between 99.1 and 99.2. Dr. Leary, however, understood “99 ½” as 99.5. To resolve the dispute, the Treasury Department sent the plate to the National Academy of Sciences, which only added to the confusion when it measured the plate at 99.05. Meanwhile, a Harvard professor decided the value was actually 99.42, and the company that had made the quartz, Schmidt & Haensch of Berlin, responded to the investigators’ queries by stating:

It seems that our number engraved on it is the correct one. [We] found it to be 99.12, and several other eminent professors came near it or had exactly the same. Only one gentleman who is in the sugar trade found it one-tenth less, *but then these gentlemen find always a little lower than others* [emphasis added] [5].

**Fig. 2:**
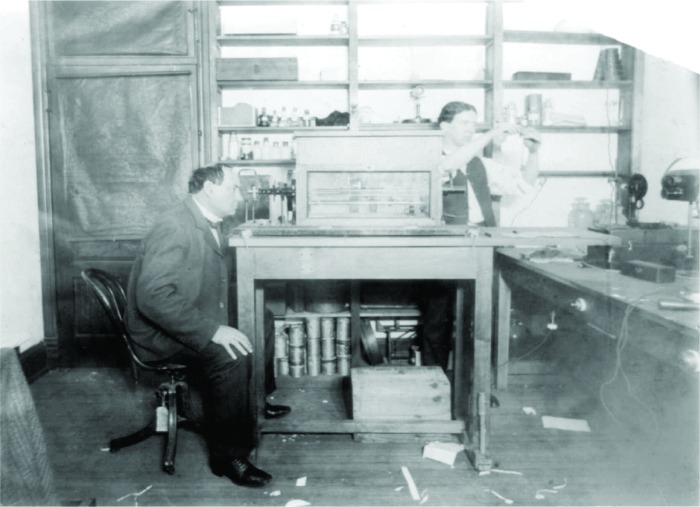
Dr. Harvey Washington Wiley peering into a saccharimeter in 1902. The box around the device protects it from outside disturbances. (Library of Congress)

In any case, the agents reported, it turned out that the plate could not be at fault after all, because Boston’s measurements were very close to those of Philadelphia. Frustrated, the agents sought the services of Dr. Harvey W. Wiley (Fig. 2), the Agriculture Department’s famed chemist. He found that New York’s readings for sugar purity were consistently more than half a percent lower than the readings everywhere else, but he could only account for a fraction of that error through differences in equipment; and in the long run, random mistakes would cancel each other out. “There is some radical defect,” wrote the puzzled Dr. Wiley, “in the method of examining sugars at the New York customs-house.”

That “radical defect” lay perilously close to outright fraud. Dr. Sherer, the agents eventually determined, was intentionally reporting the lowest measured figure to give the benefit of the doubt to the importer of the sugar. He never reported a false result, but always gave the lowest of a series of tests. Still, the agents were appalled. “We suggest that this is an improper practice,” they wrote. “A doubt is at best an unsafe standard.” When he received the report, Secretary Windom fired Sherer once more [5].

## 4. Sugar and the Founding of the Bureau

By the turn of the century, the lack of uniform and accurate saccharimetric methods had become a never-ending source of disputes between commercial interests and the government, and many millions of dollars were at stake. “Constant disputes are occurring, and I think,” said Dr. William McMurtrie, President of the American Chemical Society, “there is no place where there is a better illustration than in the custom-house and the relations between the United States customhouse and importers”^3^ [7].

Saccharimetry became one of the many problems that, to scientists and Congress, demonstrated the need for a national measurement agency. Testifying before a House committee hearing in 1900, on the proposed Bureau of Standards, McMurtrie naturally raised the question of sugar; it had once, he said, taken him four months of correspondence to settle a dispute over the calibration markings on a piece of laboratory equipment. If each customs laboratory had “a set of instruments which had been properly calibrated by a person in authority, such as this bill provides for, it would have been an easy matter to standardize the flask in question, and this difficulty would not have arisen.” Moreover, he said, it was not just devices but the theoretical grounding of saccharimetry that needed work. “The very large amount of sugar which is imported into this country would make a very great difference in the money value if there should be a variation of even so much as two-tenths of 1 per cent [7].” In fact, as the Bureau would soon demonstrate, a variation half as large could have a substantial impact.

**Fig. 3:**
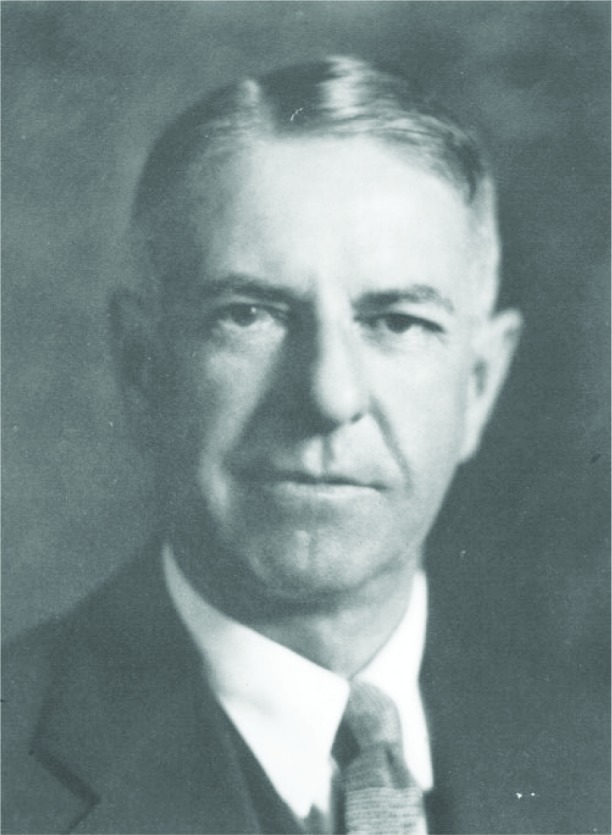
The official National Bureau of Standards portrait of Frederick Bates, taken late in his career.

Despite the importance of sugar and the other tasks before the Bureau, however, the new agency did not complete its new laboratories in northwest Washington, D.C. for a few years, so it had to wait until 1903 to begin its work on saccharimetry for the Treasury. Even then, it could only purchase a few advanced commercial saccharimeters [8]. That same year, Samuel W. Stratton, the physicist appointed as the Bureau’s first director, hired Frederick Bates (Fig. 3) to head the Polarimetry Section of the Bureau’s Optics Division. Bates was a young Kansan agricultural physicist straight out of graduate school at the University of Nebraska, where he had studied under a specialist in the optical properties of sugars. He now took charge of all of the Bureau’s saccharimetry work [9].

At about the same time, the Bureau began to test daily samples of sugar from Boston, New York, Savannah, and New Orleans, the four biggest ports of entry for sugar into the United States, and to certify the quartz plates that were used to test saccharimeters. Such close cooperation with the Customs Service continued for many years. By 1908, the Bureau was performing over a thousand analyses of sugar samples each year, and had developed the procedures that the Customs Service required its laboratories to follow. These methods, Stratton wrote, “cover the methods, instruments, and procedure, and rigidly define the scientific basis upon which the revenue is collected [8].” The Polarimetry Section continued to refine its recommended regulations for many years, to the increasing satisfaction of both the Bureau and the Customs Service. In 1912, the Treasury invited Bureau investigators on a nationwide tour of all of the Customs laboratories to recommend more substantial changes; these and later inspections imposed greater uniformity and rigor on the laboratories’ methods than did the regulations [10]. For example, in 1915 the investigators discovered, to their dismay, that Customs agents in New York City were still shaking their vials by hand to evenly distribute the contents; at the Bureau’s urging, the Assistant Secretary for Customs immediately ordered that the practice be suspended in favor of specialized shaking machines [11]. Bates, meanwhile, had demonstrated that climactic effects at different Customs laboratories could have a noticeable effect on sucrose readings [12].

The Bureau maintained particular vigilance against corruption. In 1919, Stratton advised the Customs Service to appoint an assistant chemist in the Savannah laboratory and to order the chief chemist there to exchange daily samples with New York and Philadelphia. “It is the opinion of this Bureau,” Stratton wrote, “that if the above procedure is adopted it will lessen the possible opportunities for mistake or fraud at the Port of Savannah.” Far too much money, he said, “is now collected at that port upon the polariscopic observations of only one observer, and it would seem advisible [sic] to give this revenue adequate protection by the appointment of a competent assistant chemist [13].”

From the beginning of his tenure, however, Stratton had harbored greater ambitions for the Bureau’s sugar research than simply testing samples and quartz plates. He had appointed Bates to expand the scope of the Bureau’s work, so Bates’ small team began to investigate “several theoretical problems” and started developing their own superior saccharimeter design [8].

## 5. The Overuse of Lead Acetate

Bates’ first success at NBS was precisely determining the error produced by lead acetate, the most common clarifying reagent used by sugar inspectors. In order to make a raw sugar solution more transparent, inspectors would drop a small amount of lead acetate into their sample, even though they knew it would introduce a slight error into their measurements (whether because the precipitate changed the volume of the solution, because of changes in the overall optical activity, or both). Chemists had guessed that this effect would cancel out another error introduced by slight variations in temperature. But when the International Commission for Uniform Methods of Sugar Analysis (ICUMSA) agreed on values for temperature corrections in 1900, they could not agree on similar tables for lead acetate. In upsetting this balance, wrote the chief chemist of the American Sugar Refining Company, the new corrections “aggravate, unintentionally, it is true, but nevertheless effectively, the evil resulting from the presence of the lead precipitate [14].”

In 1906, after only a few years at the Bureau, Bates decided to find the values to correct for lead acetate as well. He noted that all of the previous studies had calculated values only to tenths of degrees on the Ventzke scale, the most widely used scale for measuring sugar purity. In his research, Bates declared, “an accuracy of 0.02° Ventzke was desired.” Fortunately, he had at his disposal better equipment and better techniques than “the comparatively crude polarizing apparatus and methods” used by his predecessors^4^ [15].

**Fig. 4:**
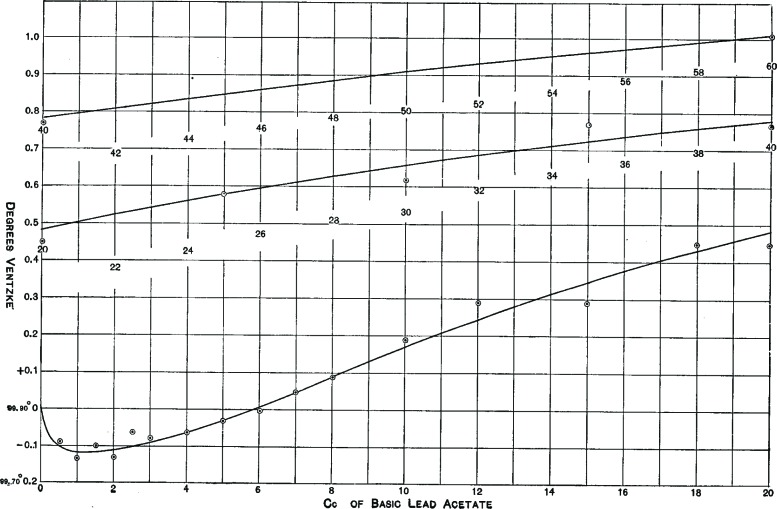
Bates’ results show the pattern of under- and over-reporting sucrose content in a sample clarified with lead acetate.

Working with J.C. Blake, over the course of several months, Bates combined samples of high-quality commercial sugar—not, he noted, chemically pure sucrose—with various amounts of lead acetate (half a cubic centimeter in some samples to 63 cc^5^ in others) and took careful polariscopic readings. Their final paper, published in the *Bulletin of the Bureau of Standards*, included a comprehensive table of the differences between precise and over-clarified polariscope readings. When Bates plotted his meticulous results (Fig. 4), he found that the reagent had much more of an effect than anyone imagined:

The curve shows beyond doubt that…basic lead acetate first causes a lowering of the polariscopic reading of sugar in solution amounting to more than 0.1° V for normal concentration, and that further addition of the same reagent causes continuous rise in the polarization up to the limit (63 cc) investigated. It will be observed that when about 6 cc of lead sub acetate are added, the polarization is not affected by the curve crossing the axis at this point [15].

After Bates and Blake published their results, handbooks on sugar analysis began to include a version of their table of errors and advised practitioners to adjust their results by a suggested amount [17]. It remained for others to find precise corrections for raw sugar, but the Bureau scientists had made a crucial contribution to the accurate determination of sucrose.

## 6. The Bates Saccharimeter in Context

Sucrose is an “optically active” molecule; that is, it rotates the direction of the polarization of light (without actually refracting the light itself). A certain concentration of an optically active substance will rotate the polarization through a known angle, per unit of distance traveled by the light. Knowing the latter, all a scientist had to do was measure the rotation of the polarization to find the concentration.

**Fig. 5:**
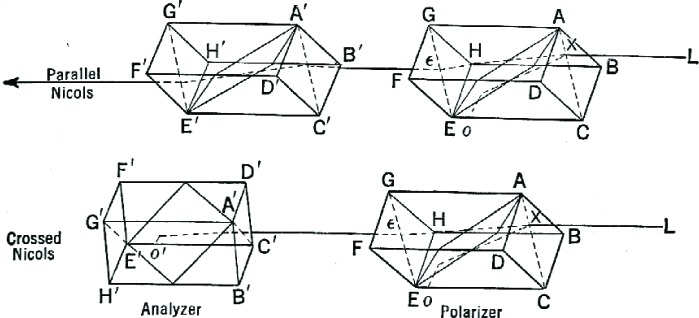
Diagram of “parallel” “crossed” nicols, from Browne and Zerban’s Physical and Chemical Methods of Sugar Analysis, 2nd ed., p. 143. In the parallel nicols the light beam is refracted away from, and then back toward, its original direction, but in the crossed system the beam is deflected out of the second nicol.

Unfortunately, making the measurement proved theoretically and practically difficult. Biot’s original polariscope of 1840 relied on a system of black mirrors to produce polarized light. Two years later, however, the German physicist Ventzke was able to produce a device which relied not on mirrors but on prisms—specifically, prisms of Nicol, or “nicols” (Fig. 5), made of Iceland spar. This proved a vastly superior principle by which to construct polariscopes.

Ventzke chose Iceland spar because its natural shape is a rhombohedron. A beam of ordinary unpolarized light incident at the proper angle, to the small face of the rhombohedra crystal, will refract into two beams, polarized at right angles to each other. Taking such a crystal, a few inches long, he bisected it perpendicularly to the small faces, then reattached the halves with Canada balsam cement. Afterwards, one of the beams was deflected out by the increased refractive index of the cement used to reattach the halves while, the other was directed back on the original path—the beam that emerged from the nicol was composed only of polarized light.

Each nicol has a plane perpendicular to the small faces and containing the optic axis, the line running between the two doubly-obtuse corners of the rhombohedron. This is called the axial plane. If two such nicols are placed such that their axial planes are parallel, all of the light emerging from the first nicol (the polarizer) will pass directly through the second (the analyzer). As the plane of the analyzer is turned away from the polarizer’s, however, less light will get through, and when they are perpendicular, none will. At that point, an observer looking through the end opposite the light source will see only darkness. This position of total darkness is called the “critical position” of the device.

An optically active material, such as a sample of sucrose or a plate of quartz, will rotate the polarized beam if the material is interposed between polarizer and analyzer. Some light will reach the eye of the observer, and the analyzer itself will need to be rotated to compensate in order to restore either the position of total light or the position of total darkness. The proper corrective will be an angle of equal magnitude but opposite direction to the rotation induced by the substance. By attaching the analyzer to a circular scale, the angle can be measured. Polarimeters, polariscopes, or saccharimeters all allowed the experimenter to read the rotation on a graduated scale which he saw as he looked through an eyepiece. In a standard polarimeter or polariscope the scale ticked off circular degrees, while in a saccharimeter the scale was calibrated especially for sucrose. Each of the many competing sucrose scales gave the percentage concentration of sucrose compared to the (essentially arbitrary) standard solution for that scale, whose rotation of light had been previously measured and recorded. Which scale a particular device used often depended on the country and date of its manufacture, and a scientist had to be sure he was using the one with which he was familiar.

This was the basic principle of the saccharimeter, but the picture was complicated (literally) by the quality of the light. Optically active materials like sucrose rotate each frequency of light through a slightly different angle. It is important, therefore, to have a relatively intense monochromatic source of light as possible; a task to which the Bureau devoted many resources during its early years [8]. Lacking such a monochromatic source, testers used a filter, a layer of potassium bichromate solution through which the light had to pass, and which could produce an excellent approximation of monochromatic light. But it was not perfect; sucrose rotated filtered light slightly less than pure monochromatic rays [18].

Another solution was to use white light to introduce another prism; this one a movable wedge made of quartz, and optically active. Moreover, quartz disperses the frequencies of light in almost exactly the same way as sucrose. By sliding the wedge, the experimenter could adjust the total thickness to counter the sucrose’s rotation and return each of the components of white light to its original position. Such a device was called a “quartz-wedge compensator.”

**Fig. 6:**
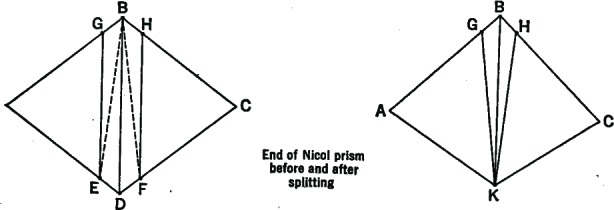
Construction of a Jellett half-shadow prism. The sections GEB and HFB were removed.

In 1862, an Irish physicist named Jellett introduced the first half-shadow prism (Fig. 6), which was modified a few years later by Cornu. The Jellett-Cornu prism was a “rhomb” (a rhombic cylinder) of Iceland spar, cut lengthwise through the shorter diagonal of the small face. A small amount was shaved off the freshly cut face of each half, then the two were reunited. Thus, the axial planes of the two halves were no longer parallel but formed a slight angle, called the “half-shadow angle.”

**Fig. 7:**
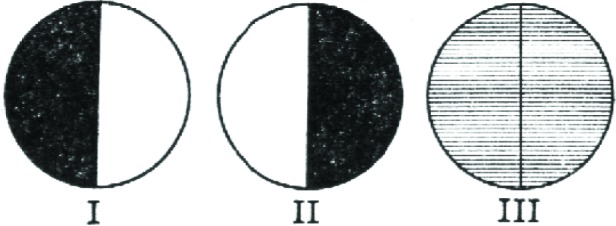
What the observer saw as he looked through a half-shadow device. In the first two positions, the light in one field is completely obscured.

**Fig. 8:**
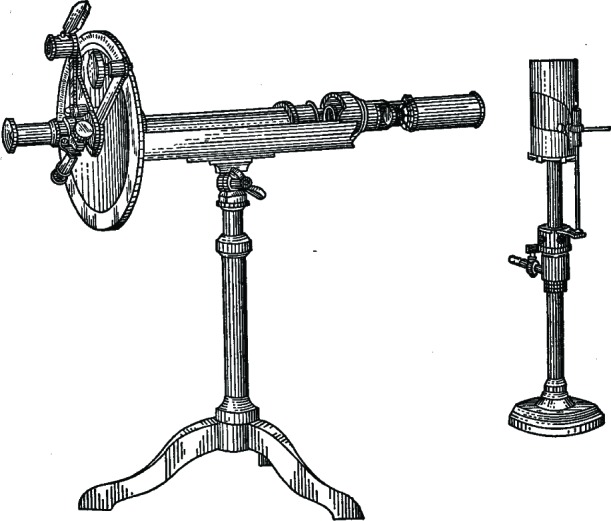
A saccharimeter relying on the Jellett-Cornu prism.

When the axial plane on one half of the Jellett-Cornu prism (Fig. 7) is perpendicular to that of the polarizer, the observer will see darkness in that half but some light on the other, and vice versa. If the bisector of the half-shadow angle is perpendicular to the polarizer, however, the light will illuminate both sides of the visible field equally. The smaller the half-shadow angle, the more sensitive the device was to small changes in optical rotation, and the more accurately saccharimetric measurements (Fig. 8) could be made. The Jellett-Cornu device could work with any kind of light, but because the prism was physically cemented together, the angle was fixed. Since smaller half-shadow angle meant that less light would reach the observer, a device constructed for extremely high accuracy would not also work for darker sugar solutions [18].

**Fig. 9:**
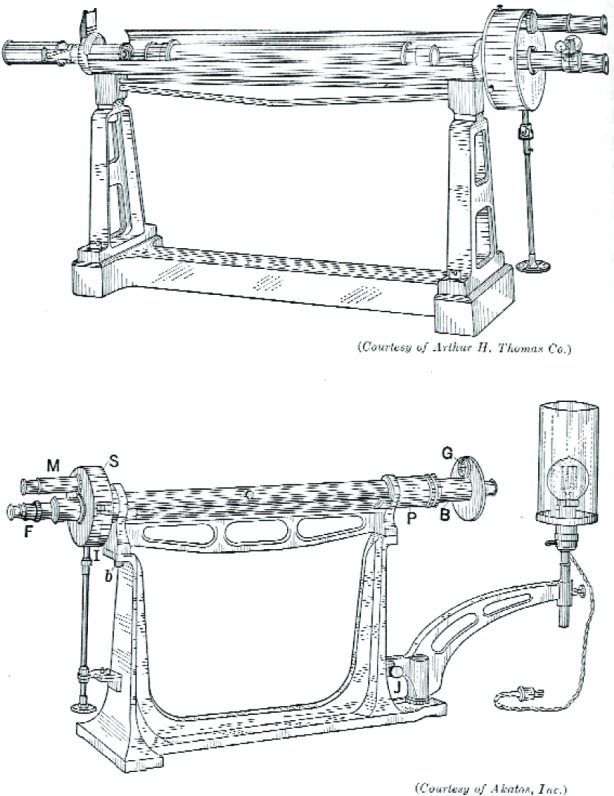
Laurent and Lippich saccharimeters.

In 1877, in an attempt to resolve this issue, the French physicist Laurent (Fig. 9) developed another kind of half-shadow device which relied on pieces of quartz machined extremely precisely to half of a wavelength of the light. In this case, it was the polarizer which rotated through a small angle. The experimenter could adjust the sensibility of Laurent’s device, but without a compensator it could only be used with monochromatic light. Finally, in 1880, the Austrian physicist Lippich introduced a more accurate saccharimeter whose half-shadow angle was adjustable and also to, with a quartz compensation system, use unfiltered white light. But the Lippich device, too, came with a caveat—because it was inherently asymmetrical, every time the half-shadow angle was changed, the zero point of the saccharimeter moved and had to be recalibrated. This posed no problem for the expertly trained, but was an issue for less-skilled customs inspectors, on whose tests the value of so much commerce rested.^6^

At the turn of the century a number of manufacturers, such as Schmidt & Haensch of Berlin, Bellingham & Stanley of London, and the brothers Josef & Jan Fric of Prague, all produced saccharimeters following one or more of the Jellett-Cornu, Laurent, or Lippich designs. Each company worked to develop unique means of lighting the circular scales and reducing error by making the rotation of the nicols and the sliding of the quartz wedges as smooth as possible. But no one had yet developed a method for uniting the Lippich system—which gave the best results—with a quartz compensator, and still allow it to have a sensibility that could easily and reliably be altered to suit the circumstances of each particular test. This was the task that Bates set for himself.

In his paper of January, 1908, Bates published the results of his investigations. He began by reviewing the three types of half-shadow saccharimeters, lamenting that “the greatest weakness has been the lack of an adjustable sensibility. Only one value of *a* [the half-shadow angle] can be used and it must necessarily be large enough to give sufficient light to read, for example, the darkest colored raw sugar solutions.” A tester was hamstrung by the need to err on the side of light over sensibility. “If then it were possible to retain the white-light source and at the same time have *a* adjustable,” he wrote, “a distinct advantage in polariscope construction would be made [19].”

**Fig. 10:**
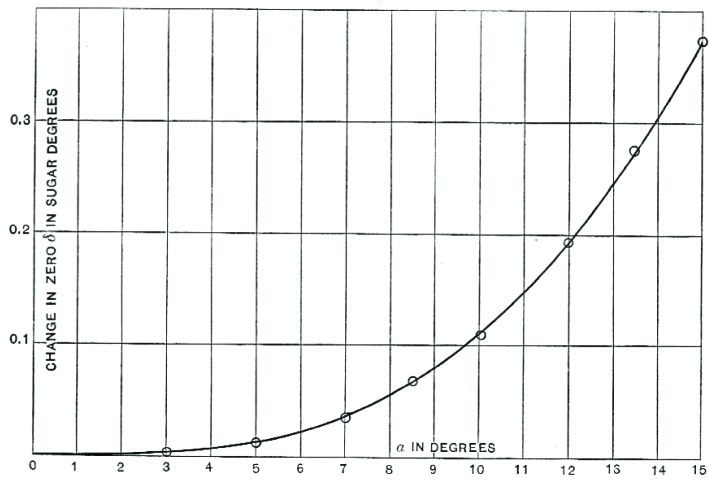
The relationship in a Lippich device between the size of the half-shadow angle a, and the displacement of its bisector from zero, as plotted by Bates.

**Fig. 11:**
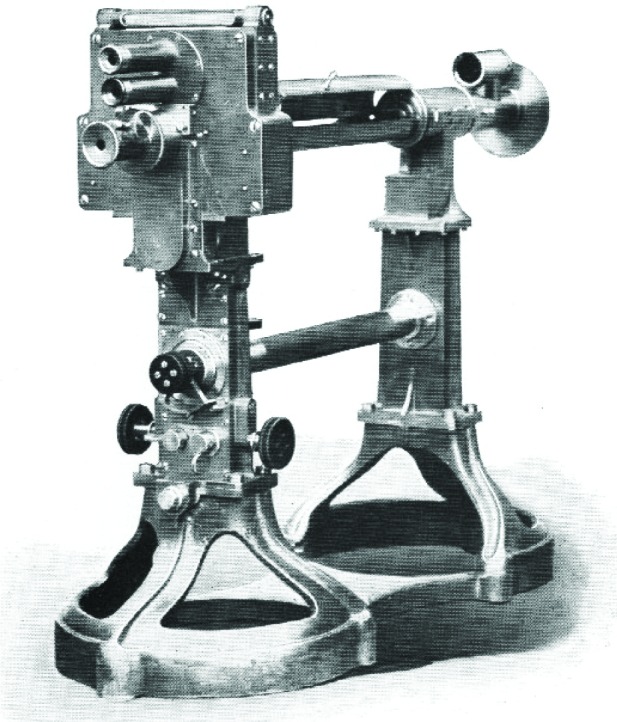
Bates’ saccharimeter.

He then proceeded to demonstrate, mathematically, the relationship between the modification of the half-shadow angle and the displacement of the zero point on the scale, as a result of that modification (Fig. 10). Unfortunately, his proof concluded in a formula that related the tangent of one angle to that of another, times a coefficient including the square root of the ratio of the intensities of the light in both halves of the observer’s view. “It would seem a difficult task,” he suggested, “to build a mechanism that would…satisfy the theoretical value of” the displacement. But Bates then plotted a curve of the value of the displacement against the value of *a*, and he knew how to construct a mechanism that would follow this curve, with an error no greater than one tenth of one degree; though this time he measured the variation on the International Sugar Scale. “The instrument shown (Fig. 11),” he announced, “was built for the Bureau of Standards to fulfill the theoretical conditions mentioned above.”

Bates had devised a system of gears and milled heads that he designed to fit onto a Fric saccharimeter using the Lippich system. He also included a thermometer to indicate the temperature of the quartz wedges, which had been the source of some error in previous devices. The gears could be locked “instantly” by clamps, and included smaller screws for especially fine adjustment [19]. The only disadvantage of Bates’ device was that its especially fine construction made it far more expensive. The Fric catalog listed the price at between $800 and $900, depending on its capacity, which was four times the price of any Schmidt & Haensch instrument [9]. Yet its overwhelming virtues made it an instant success. In the same year that it was developed it was adopted as the standard saccharimeter for the United States Customs Service [8]. This design was produced, with slight but continual modifications, for several decades.

Bates’ accomplishment made it possible for all sugar testers—from customs assistants in minor ports of entry, to skilled scientists in the best-equipped laboratories—to detect sucrose to an equally high degree of accuracy. Within a few years, Stratton reported that as a result of the Bates saccharimeter’s introduction, “the differences in the results at the five principal sugar ports have been reduced to as low as 0.2 per cent; a concordance which is quite satisfactory [8].” Bates’ device was so successful that it remained the standard Customs saccharimeter through the nineteen-forties [18].

## 7. The International Commission and the One-Hundred-Degree Point

Bates made his most lasting accomplishment and most remarkable scientific achievement, however, when he solved a problem that had vexed the Bureau from the very beginning: “the determination of the 100° Ventzke point on the saccharimeter scale,” as Director Stratton wrote in his 1910 report. (Stratton may have been confused here. Bates was now working on the Herzfeld-Schönrock scale, which was a slightly modified version of the scale the International Commission had adopted in 1900, and not the Ventzke scale itself. His error is forgivable: definitions of scales were notoriously confusing until the nineteen-thirties [21].)

To refine this measurement Bates’ laboratories needed a steady supply of exceedingly pure sucrose, which the Bureau provided by 1908, using recrystallization in a vacuum. The Bureau also had to satisfy commercial demand for pure sucrose; businesses wanted it to test their own saccharimeters (and calorimeters, since these standard sugar samples were also used to measure the heat content of other fuels). In fact, sucrose was one of the Bureau’s earliest Standard Reference Materials (number 17) [8]. “The distribution of such samples for standardization purposes marks an important step in optical measurements and calorimetry,” wrote Stratton.

At first, the scale problem seemed one only of refining the existing measurement to an ever-increasing degree of precision. By 1910, however, Bates realized that the Herzfeld-Schönrock scale was off by a substantially greater amount than he had suspected. To the Bureau, this issue became “the most important problem at present in saccharimetry [8].” At Columbia University, during the seventh meeting of the International Commission in 1912, Bates announced a startling discovery—the 100° point on the scale was one-tenth of one degree too high. A solution produced according to the standards the Commission adopted in 1900 would read, not 100°, but 99.895° when tested properly. This seemingly small error had cost the United States government half a million dollars in lost tariff revenue in the previous decade [8].

The ICUMSA, at Bates’ recommendation, appointed a special committee to determine the most precise value possible and make recommendations. In addition, the Commission named the Bureau’s star scientist as chairman. Moreover, while the members of the committee were drawn from both the Austrian and German Sugar Institutes, as well as the Physikalisch-Technische Reichsanstalt (the German standards-setting agency) in Berlin, it was formally agreed that the Bureau’s representatives should lead the effort [8].

This committee was to report back shortly with recommendations for fixing the sugar scale. Unfortunately, the members of the committee soon found themselves behind opposing fronts in the Great War. As Stratton noted in his 1915 annual report, “Owing to the present conditions abroad, no cooperative work has so far been attempted [8].” Undeterred, Bates persisted alone, and in 1916 he concluded that the sugar community faced two choices—to maintain the standard sample mass of sugar at 26 grams (in 100 cm^3^, at 20°C, measured in a 200 mm tube) and change the value of the scale, or change the standard mass to 26.026 g and keep the scale the same. By 1917, it had become clear that the committee would not meet to approve a new standard any time soon, and Stratton and Bates decided that there was no point in “standardizing sugar-testing apparatuses on a basis now known to be in error [8].” American customs laboratories began using saccharimeters adjusted to the corrected scale, despite the complaints of sugar scientists that it had not been approved by the ICUMSA; many other manufacturers and laboratories followed suit [9].

The International Commission, however, became another casualty of the Great War. Its communications severed by the fighting, the Commission ceased to function for the next two decades, during which time many of its members passed away, including the president and secretary. Bates worked tirelessly to revive the organization, but it was not until September of 1932 that he could convene ICUMSA’s eighth session at the University of Amsterdam—the city whose merchants had developed the color standard that Congress had adopted in 1861. After electing Bates as president, the Commission set about repairing the sugar scale.

First, the Commission decided to create a new scale, optimistically called, again, the International Sugar Scale (ISS), measured in degrees S, or sugar. The ISS would use the existing weight of 26 g, but adjust the 100° point to agree with Bates’ findings [22]. However, since so many expensive instruments were already calibrated in the Ventzke scale, and since experimenters had become accustomed to its use, the ICUMSA did not throw out the old scale entirely. Instead, it provided for another standard weight of 26.026 g, which would be produced in hexagonal cylinders so that scientists could readily distinguish it from the cubical 26 g weights; the standard ISS weights [18]. It proved impossible to keep replacing expensive saccharimeters every time the previous scale was discredited. Absolute and ideal global standardization, in other words, necessarily took a back seat to the requirements of commerce.

In the Director’s Annual Report for 1933, Lyman J. Briggs wrote:

*International sugar scale*.—the eighth session of the International Commission for Uniform Methods of Sugar Analysis at Amsterdam in September 1932 officially adopted the Bureau’s proposed scale for the buying and selling of sugar throughout the world.^7^

Unfortunately, the new system never became as universal as the ICUMSA hoped it would, though it did spread as manufacturers decided to incorporate the ISS into their equipment after 1932. Experimenters and testers had to be absolutely certain that they were using the right weights for their scale and their device. But as the prominent sugar chemist C. A. Browne noted, “It does not really matter what scale is used, as long as the normal weight solution gives a reading of 100 [18].” As Bates demonstrated, getting the normal weight to read 100 on any scale was a problem that demanded the utmost scientific ingenuity, instrumental quality, experimental rigor, and institutional support.

## 8. Conclusion

In 1942, Bates was named chief of the Optics Division. That year, the Bureau published its Circular 440, “Polarimetry, Saccharimetry, and the Sugars,” an eight-hundred-page book that compiled all of the research that Bates and the other sugar researchers had conducted over the previous four decades [21]. By 1958, Bates had served three terms as the head of the ICUMSA and had been elected its Honorary Lifetime President. At its convention in Washington, D.C., the members dedicated the session’s publications to Bates and gave him a standing ovation.

The Bureau of Standards played a crucial role in the scientific and commercial development of international sugar standards. Its scientists, led brilliantly by Bates, overcame the disadvantages of working in a new, under-funded, and under-equipped institution to conduct pioneering research across the entire field of saccharimetry. Theirs were among the earliest successes for the fledgling Bureau. Moreover, in saccharimetry the Bureau could from its first efforts fulfill its dual missions of aiding commerce and the government, through its routine collaboration with both industry and the Customs Service, as well as by its remarkable scientific advances, both of which helped to resolve longstanding problems with the way sugar tariffs were levied in the United States. Yet, the Bureau did not make those advances in a vacuum. On the contrary, perhaps the most impressive aspect of the Bureau’s work in saccharimetry was that it conducted research that contributed so quickly, and in such novel and remarkable ways, to further a robust scientific discipline.

## Footnotes

1 The information in this section is largely thanks to Deborah Jean Warner, curator of the physical sciences collection at the National Museum of American History, and her unpublished article “How Sweet it Is: Sugar, Science, and the State” (April 6, 2006 revision, with endnotes).

2 A “saccharimeter" is a polarimeter specifically calibrated for measuring sugar, but there are no substantial differences. Whether or not the device should be called a "meter" at all has been the subject of some disagreement, as neither actually measures the polarization directly, so some sources call the device a "polariscope" (though never a "sacchariscope") to indicate the more ambiguous nature of the measurement. All three terms are used in this paper, but the device is the same.

3 In the *Congressional Record* he is identified as “M’Murtrie” but according to the American Chemical Society the proper spelling is “McMurtrie.”

4 Bates published his results in the Ventzke scale, even though ICUMSA’s recommendations in 1900 made the scale officially obsolete.

5 Bates used “cc” but the modern SI system denotes this unit of volume by “cm^3^”.

6 Ibid., 154. Testers disagreed on how serious a problem this readjustment presented. The writer of one textbook, Noel Deerr, mocked his fellow chemist Landolt, who “asserts that with technical instruments a fixed half-shadow angle should be employed, since with every change of angle there is a change in the zero which requires adjustment. While this reasoning may be correct with regard to chemists of a certain mental type, it is quite inapplicable to others of a superior intelligence” (Deerr, 485).

7 Annual Report of the Director of the Bureau of Standards, 1933: 53. The name “International” was an unfortunate choice: Sidersky and Pellet had already proposed an “international” scale at the Second International Congress of Applied Chemistry in 1896. Based on a normal weight of 20 grams, theirs was also called the “bidecimal system,” reflecting the intentions of the two chemists that calculations would benefit from the weight’s easy divisibility. Unfortunately, changes in the original French standard on which the bidecimal system was based eventually negated these advantages by lowering the required weight to 19.973 grams (Browne and Zerban, *Sugar Analysis*, 181).
